# Impact of diagnosis‐to‐ablation time on clinical outcomes in patients with early‐onset atrial fibrillation

**DOI:** 10.1002/clc.24194

**Published:** 2023-12-06

**Authors:** Le Zhou, Yu Kong, Caihua Sang, Shijun Xia, Chao Jiang, Liu He, Xueyuan Guo, Wei Wang, Songnan Li, Chenxi Jiang, Nian Liu, Ribo Tang, Deyong Long, Xin Du, Jianzeng Dong, Changsheng Ma

**Affiliations:** ^1^ Department of Cardiology Beijing Anzhen Hospital Capital Medical University, National Clinical Research Center for Cardiovascular Diseases, Office of Beijing Cardiovascular Diseases Prevention Beijing China; ^2^ Heart Health Research Center Beijing China; ^3^ Cardiovascular Diseases University of New South Wales Sydney Australia

**Keywords:** atrial fibrillation, diagnosis‐to‐ablation time, radiofrequency ablation

## Abstract

**Background:**

Evidence was lacking for the early choice of radiofrequency ablation (RFA) among patients with early‐onset atrial fibrillation (AF).

**Hypothesis:**

This study aimed to explore whether earlier RFA was associated with better clinical outcomes among early‐onset AF patients.

**Methods:**

Patients, who were diagnosed with AF before 45 years and underwent their first RFA procedures at baseline of the China Atrial Fibrillation registry, were enrolled and divided into four diagnosis‐to‐ablation time (DAT) groups: DAT ≤ 1 year, 1 year < DAT ≤ 3 years, 3 years < DAT ≤ 6 years, and DAT > 6 years. Another group of nonablation patients, who were newly diagnosed with AF and younger than 45 years, were also included. Adjusted associations of groups with composite cardiovascular events (cardiovascular death, embolism, major hemorrhages, or cardiac rehospitalization) or recurrent AF were analyzed using Cox proportional hazards models.

**Results:**

Among 1694 patients who underwent their first RFA at enrollment, incidences of composite cardiovascular outcomes were increasing with extension of DAT (DAT ≤ 1 year: 6.1/100 person‐years, 1 year < DAT ≤ 3 years: 7.9/100 person‐years, 3 years < DAT ≤ 6 years: 7.6/100 person‐years, DAT > 6 years: 10.5/100 person‐years; *p* < .001). In comparison with DAT > 6 years group, the DAT ≤ 1 year group was associated with reduced risk of cardiovascular events (adjusted hazard ratio, HR [95% confidence interval, CI] = 0.64 [0.47–0.87], *p* = .005) and AF recurrence (adjusted HR [95% CI] = 0.70 [0.57–0.88], *p* = .002). Associations remained similar after stratified by AF types. Compared to nonablation group (*n* = 413), DAT ≤ 1year patients tended to show lower cardiovascular risk (adjusted HR [95% CI] = 0.78 [0.58–1.05], *p* = .099) and lower risk of recurrent AF (adjusted HR [95% CI] = 0.46 [0.38–0.55], *p* < .001).

**Conclusions:**

A shorter DAT was associated with a lower risk of cardiovascular events and recurrent AF for early‐onset AF patients.

## Graphical abstract

Impact of diagnosis‐to‐ablation time (DAT) on clinical outcomes in patients with early‐onset atrial fibrillation (AF).

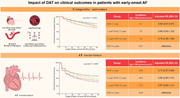

## 1 INTRODUCTION

Atrial fibrillation (AF) used to be known as an age‐related arrhythmia, which is more prevalent in older patients.^1^ However, early‐onset AF is becoming increasingly common in recent years.^2^ The general notion often is that the relative contribution of heritability may be of greater importance in younger patients, of whom a higher proportion had familial AF.^3^ Recently, many studies have showed the earlier rhythm‐control therapy was taken, the better prognosis would be.^4^, ^5^, ^6^, ^7^, ^8^ From the EAST‐AFNET 4 trial, early rhythm‐control therapy was shown to be associated with a lower risk of cardiovascular outcomes than usual care among patients with new‐diagnosed AF (diagnosed ≤ 1 year before enrollment).^4^ Other observational studies also found that shorter diagnosis‐to‐ablation time (DAT) improved the success rate of radiofrequency ablation (RFA).^5^, ^6^, ^7^, ^8^, ^9^, ^10^ Nevertheless, despite the Guidelines do not distinguish medical management between early‐onset AF patients and the general patient population,^1^ most existing medical evidence is derived from studies that enrolled patients older than 60 years.^4^, ^5^, ^6^, ^7^, ^8^, ^9^, ^10^ For early‐onset AF patients, who were underrepresented in AF trials and whose myocardial structural and electrophysiological changes might be different from others,^11^ evidence is still lacking for early choice of interventional therapy. This study aimed to explore whether earlier RFA was associated with lower cardiovascular risk and AF recurrence risk among early‐onset AF patients.

## 2 METHODS

### 2.1 Subjects and data collection

The participants in this study were enrolled from the China Atrial Fibrillation (China‐AF) Registry, which is a prospective, multicenter, hospital‐based registry that recruited 31 tertiary and nontertiary hospitals located in Beijing, and Anzhen Hospital, having the largest Arrhythmia Center in China, was the main site that doing RFA procedures for AF patients.^12^ Even though this is a retrospective analysis, but it prospectively collected registry data. Patients, who had been first diagnosed with AF between 18 and 45 years, underwent their first RFA procedures at the baseline enrollment of China‐AF from August 2011 to December 2020, and had been followed up for at least 6 months, were included in this study. RFA is an effective method for controlling and terminating AF. Furthermore, we included a control group of nonablation patients, who were newly diagnosed with AF (within 1 year) and younger than 45 years at enrollment. Among all participants, those patients with moderate/severe mitral stenosis and those with mechanical prosthetic heart valve(s) from other patients with AF were excluded.

Clinical data at baseline enrollment of China‐AF and patients' characteristics were captured via a web‐based electronic data capture system in the well‐designed form of electronic case report forms, including demographic information (sex, age at the first diagnosis of AF, age at the first RFA, body mass index [BMI], estimated glomerular filtration rate [eGFR]), DAT, type of AF, comorbidities (hypertension, diabetes, hyperlipidemia, congestive heart failure, thromboembolism, vascular disease, major bleeding, hypertrophic cardiomyopathy, dilated cardiomyopathy, obstructive sleep apnea‐hypopnea syndrome), results of echocardiography (left ventricular ejection fraction [LVEF], anteroposterior diameter of left atrium [LA]), concomitant medication (Statins, angiotensin‐converting enzyme inhibitors/angiotensin receptor blockers, oral anticoagulants [OAC]), CHA2DS2‐VASc, perioperative complications (thromboembolism, bleeding complications, atrioventricular block, sinus node injury, cardiac tamponade, pulmonary vein [PV] stenosis, pneumothorax or hemopneumothorax, left atrioesophageal fistula), alcohol assumption, and smoking, and so forth, were collected. Patients with a CHA_2_DS_2_‐VASc score of 2 or more in males or 3 or more in females should be on anticoagulation unless contraindicated.

Definitions of each variable were consistent with the American College of Cardiology/American Heart Association recommendation on AF clinical data standards.^13^ The population who received ablation therapy was divided into four groups according to different intervals of DAT: DAT ≤ 1 year, 1 year < DAT ≤ 3 years, 3 years < DAT ≤ 6 years, and DAT > 6 years. The nonablation group could be compared with the DAT ≤ 1 year group. All enrolled patients were followed up by face‐to‐face clinical interviews or telephone interviews at the third and sixth months since enrollment, and every 6 months after that. If at any time during the follow‐up, patients experienced symptoms or irregular heartbeat suggesting arrhythmia recurrence and went to any hospital for examination, the reports of 12‐lead electrocardiogram (ECG), 24‐h Holter, or implantable loop recorder (ILR) would be collected by our colleagues, and additional monitoring would be performed. Any arrhythmia episode would be recorded after reviewing previously mentioned records. Other information on death and cardiovascular events that occurred during follow‐ups was also collected and confirmed at each visit.

This study was approved by the ethics committee of each institution's review board, and written informed consent was obtained from each patient. Beijing Anzhen Hospital was responsible for study coordination and site management.

### 2.2 Endpoint outcomes

The endpoint outcomes of this study were composite cardiovascular events (cardiovascular death, embolism, major hemorrhages, or cardiac rehospitalization) or recurrent AF. Cardiovascular death was defined as death due to any cardiovascular cause, such as malignant arrhythmias, heart attacks, and so forth. Cardiac rehospitalization was collected during follow‐ups if there was any hospitalization record with a primary discharge diagnosis related to heart disease, including acute coronary syndrome, heart failure, arrhythmia, and so forth. Embolism in this study included ischemic stroke (documented stroke or cerebrovascular accident consisting of acute loss of neurological function caused by an ischemic event with residual symptoms at least 24 hours after onset) and peripheral embolism (abrupt vascular insufficiency associated with clinical and radiological evidence of arterial occlusion in a vascular bed other than the cerebrovascular system). Major hemorrhages were defined as bleeding into or around the brain, or bleeding led to a fall in hemoglobin ≥ 20 g/L, or transfusion of ≥2 units of whole blood or red blood cells. Patient‐reported nonfatal cardiovascular outcomes were adjudicated by two independent clinicians separately. Disagreement was resolved by discussion or by involving other senior clinicians. Postoperative AF recurrence was defined as any episode of atrial tachyarrhythmia (AF, atrial flutter [AFL], atrial tachycardia) if it lasted for ≥30 seconds and was documented by ECG, Holter, or ILR outside the 3‐month postablation blanking period.^14^

### 2.3 Catheter ablation procedure

All patients enrolled in this registry underwent bilateral circumferential pulmonary vein isolation (PVI) during the catheter ablation procedure. Utilizing the CARTO 3 system from Biosense Webster, we conducted a three‐dimensional electroanatomical mapping. Ablation was carried out using a standard 3.5 mm irrigated catheter. For patients diagnosed with persistent AF, cardioversion was initiated to reestablish sinus rhythm if AF wasn't resolved after the 2C3L protocol (“2C3L” strategy^15^). During sinus rhythm, the PVI and linear block were confirmed. The approach for detecting and eradicating non‐PV triggers entailed: (1) Zeroing in on the primary beat that initiated AF; (2) systematic pacing from the right atrium for 10 seconds, using a cycle length of 200–300 ms; (3) administering a high dose of isoproterenol for 10–15 minutes at a rate of 20–30 mg/min. In instances where triggering events emerged naturally or through induction techniques, we proceeded with further mapping and ablation. For paroxysmal AF, if the patients were diagnosed with AFL before the procedure, cavotricuspid isthmus linear ablation would be performed. Additional ablations, if needed, were performed at the operator's discretion. The endpoint of the procedure was pulmonary isolation and bidirectional block of ablation lines.

### 2.4 Statistical analysis

Categorical variables were expressed as percentages or frequencies and compared using the *χ* ^2^ test or Fisher's exact test among different DAT groups, while continuous variables were expressed as means ± standard variances or median (interquartile range [IQR]), depending on whether the data were normally distributed, and compared using one‐way analysis of variance test or Kruskal–Wallis test among groups.

Time‐to‐first‐event analyses were used for clinical outcomes. Kaplan–Meier estimation was used to construct survival curves and log‐rank tests were performed to determine the statistically significant difference in survival rates that are free from composite endpoints or AF recurrence among groups. Univariate and multivariate Cox proportional hazard regressions were carried out to calculate the hazard ratios (HRs) and their 95% confidence intervals (CIs) of different groups and all possible influence factors of composite endpoints or AF recurrence and to estimate the modified effects of DAT intervals on previous outcomes across different AF types and year of enrollment. Since there were 8.1%, 2.7%, and 19.5% missing data of BMI, eGFR, and anteroposterior diameter of the LA in ablation patients (*n* = 1694), while 6.5%, 33.2%, and 31.0% in nonablation patients (*n* = 413), respectively, multiple imputations using the approximate Bayesian bootstrap hot‐deck imputation method were applied through PROC MIANALYZE procedure during the process of building Cox regression models, HRs, and their 95% CIs were inferred from 20 imputed data sets.

All tests were two‐sided, and *p* < .05 was considered statistically significant. Data were analyzed using SAS software version 9.4 (SAS Institution Inc.).

## 3 RESULTS

In the ablation group, 1694 patients, among whom AF initiated before 45 years old, were ultimately enrolled, with a mean age of 38.2 ± 5.9 years at their first diagnosis of AF and a mean age of 44.4 ± 8.9 years at their first RFA procedures. The median age of 413 nonablation patients, who were newly diagnosed with AF, was 39.0 years at enrollment. The median age for the four RFA groups was 40.0, 39.7, 40.2, and 39.3 years old at the time of AF diagnosis, and 40.0, 41.0, 45.0, and 47.0 years old at the time of the first AF ablation. The range of age at the time of their first RFA was from 18 to 80 years old. Higher proportions of hypertension, diabetes, hyperlipidemia, thromboembolism, vascular disease, and high‐stroke‐risk patients were presented in patients with DAT > 6 years (All *p* < .05), while the OAC treatment rate before enrollment was also higher among those patients (*p* < .001) (Table 1).

**Table 1:** Comparison of baseline characteristics among patients with early‐onset AF by different DAT intervals.

Characteristics	Control group	RFA groups				*p* Value
	Nonablation (*N* = 413)	DAT ≤ 1 year (*N* = 544)	1 year < DAT ≤ 3 years (*N* = 274)	3 years< DAT ≤ 6 years (*N* = 274)	DAT > 6 years (*N* = 602)	
Female, *n* (%)	91 (22.0)	104 (19.1)	45 (16.4)	35 (12.8)	108 (17.9)	.034
Age at the first diagnosis of AF, mean ± STD, years	36.4 ± 7.1	38.6 ± 5.6	38.3 ± 5.7	38.6 ± 5.9	37.6 ± 6.1	<.001
Age at the first RFA, mean ± STD, years	–	38.8 ± 5.6	40.2 ± 5.8	43.0 ± 5.9	52.0 ± 8.3	<.001
BMI, mean ± STD, kg/m^2^	25.8 ± 4.0	25.8 ± 4.2	26.4 ± 4.6	26.1 ± 3.3	26.1 ± 3.4	.206
eGFR, median (IQR), mL/min/1.73 m^2^	131.0 (113.5, 148.0)	125.3 (110.0, 145.5)	124.5 (110.8, 139.7)	120.7 (107.3, 136.7)	115.6 (102.2, 129.2)	<.001
Current smoking, *n* (%)	74 (17.9)	110 (20.2)	53 (19.3)	50 (18.3)	113 (18.8)	.697
Current drinking, *n* (%)	74 (17.9)	96 (17.7)	55 (20.1)	51 (18.6)	104 (17.3)	.890
Persistent AF, *n* (%)	86 (20.8)	181 (33.3)	110 (40.2)	124 (45.3)	282 (46.8)	<.001
Medical history, *n* (%)						
Hypertension	113 (27.4)	152 (27.9)	91 (33.2)	92 (33.6)	263 (43.7)	<.001
Diabetes	36 (8.72)	40 (7.35)	28 (10.2)	29 (10.6)	96 (16.0)	<.001
Hyperlipidemia	43 (10.4)	63 (11.6)	44 (16.1)	44 (16.1)	127 (21.1)	<.001
Congestive heart failure	33 (7.99)	28 (5.15)	15 (5.47)	13 (4.74)	37 (6.15)	.338
Thromboembolism	11 (2.66)	11 (2.02)	4 (1.46)	16 (5.84)	55 (9.14)	<.001
Vascular disease	7 (1.69)	5 (0.92)	2 (0.73)	7 (2.55)	36 (5.98)	<.001
Major bleeding	5 (1.21)	6 (1.10)	5 (1.82)	10 (3.65)	20 (3.32)	.023
Hypertrophic cardiomyopathy	4 (0.97)	9 (1.65)	8 (2.92)	2 (0.73)	4 (0.66)	.056
Dilated cardiomyopathy	6 (1.45)	9 (1.65)	3 (1.09)	3 (1.09)	5 (0.83)	.765
OSAHS	5 (1.21)	3 (0.55)	4 (1.46)	2 (0.73)	8 (1.33)	.630
Echocardiographic parameters						
LVEF, median (IQR), %	63.0 (57.0, 67.0)	62.0 (59.0, 66.1)	63.0 (59.0, 67.0)	63.0 (57.0, 67.0)	63.0 (60.0, 68.0)	.061
Anteroposterior diameter of LA, median (IQR), mm	37.0 (32.0, 42.0)	39.0 (35.0, 42.0)	38.0 (35.0, 42.0)	39.0 (35.0, 43.0)	40.0 (36.0, 43.0)	<.001
Concomitant medication, *n* (%)						
Statins	52 (12.6)	76 (14.0)	55 (20.1)	50 (18.3)	144 (23.9)	<.001
ACEIs/ARBs	48 (11.6)	81 (14.9)	54 (19.7)	46 (16.8)	150 (24.9)	<.001
OAC before enrollment						
OAC	37 (8.96)	74 (13.6)	43 (15.7)	55 (20.1)	136 (22.6)	<.001
Warfarin	34 (8.23)	60 (11.0)	39 (14.2)	47 (17.2)	123 (20.4)	<.001
NOACs	4 (0.97)	15 (2.76)	6 (2.19)	10 (3.65)	15 (2.49)	.209
AADs	72 (17.4)	223 (41.0)	127 (46.4)	148 (54.0)	319 (53.0)	<.001
CHA_2_DS_2_‐VASc ≥2 (male) or ≥3 (female), *n* (%)	41 (9.93)	47 (8.64)	23 (8.39)	36 (13.1)	149 (24.8)	<.001

Abbreviations: AADs, antiarrhythmic drugs; ACEI, angiotensin‐converting enzyme inhibitors; AF, atrial fibrillation; ARBs, angiotensin receptor blockers; BMI, body mass index; DAT, diagnosis‐to‐ablation time; eGFR, estimated glomerular filtration rate; IQR, interquartile range; LA, left atrium; LVEF, left ventricular ejection fraction; NOACs, new oral anticoagulants; OAC, oral anticoagulants; OSAHS, obstructive sleep apnea‐hypopnea syndrome; RFA, radiofrequency ablation.

**Figure 1:**
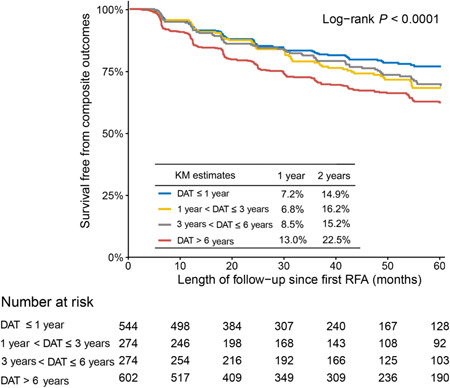
Kaplan–Meier (KM) curves for composite outcomes since the first RFA among patients with early‐onset AF by different DAT intervals. Composite outcomes: cardiovascular death, embolism, major hemorrhages, or cardiac rehospitalization. AF, atrial fibrillation; DAT, diagnosis‐to ablation time; RFA, radiofrequency ablation.

During a median follow‐up of 3.45 (IQR: 1.50–4.93) years, the incidence of composite outcomes since the first RFA increased with the extension of DAT (DAT ≤ 1 year: 6.1 per 100 person‐years, 1 year < DAT ≤ 3 years: 7.9 per 100 person‐years, 3 years < DAT ≤ 6 years: 7.6 per 100 person‐years, DAT > 6 years: 10.5 per 100 person‐years; *p* < .001). The number of patients with each cardiovascular event during the follow‐up period is shown in Table 2. Kaplan–Meier estimates of composite outcomes were compared among four groups (Figure 1), which showed higher event‐free survival probability in the shorter DAT group (log‐rank *p* < .001). The result of the multivariate Cox model demonstrated a significant association of DAT ≤ 1 year with lower risk of the composite event compared to the DAT > 6 years group (DAT ≤ 1 year: HR = 0.64, 95% CI: 0.47–0.87, *p* = .005; 1 year < DAT ≤ 3 years: HR = 0.86, 95% CI: 0.62–1.20, *p* = .376; 3 years < DAT ≤ 6 years: HR = 0.78, 95% CI: 0.58–1.05, *p* = .106) (Table 3).

**Table 2:** Number of patients who experienced cardiovascular events during follow‐ups.

	Total (*N* = 2107)	Control group	RFA groups			
		Nonablation (*N* = 413)	DAT ≤ 1 year (*N* = 544)	1 year < DAT ≤ 3 years (*N* = 274)	3 years < DAT ≤ 6 years (*N* = 274)	DAT > 6 years (*N* = 602)
Composite outcomes,^a^ *n*	520	94	96	68	71	191
Cardiovascular death, *n*	14	7	1	1	2	3
Embolism, *n*	23	5	5	4	0	9
Major hemorrhages, *n*	12	2	4	2	0	4
Cardiac rehospitalization, *n*	471	80	86	61	69	175
Recurrent AF, *n*	1075	260	207	121	146	341

Abbreviations: AF, atrial fibrillation; DAT, diagnosis‐to‐ablation time; RFA, radiofrequency ablation.

^a^ Cardiovascular death, embolism, major hemorrhages, or cardiac rehospitalization.

**Table 3:** Adjusted associations of DAT intervals with composite outcomes and AF recurrence since the first RFA among patients with early‐onset AF.^a^

Variables	Multivariable analysis for composite outcomes		Multivariable analysis for AF recurrence	
	HR (95% CI)	*p* Value	HR (95% CI)	*p* Value
DAT intervals				
DAT > 6 years	Reference	–	Reference	–
3 years < DAT ≤ 6 years	0.78 (0.58–1.05)	.106	0.97 (0.80–1.21)	.816
1 year < DAT ≤ 3 years	0.86 (0.62–1.20)	.376	0.80 (0.63–1.01)	.063
DAT ≤ 1 year	0.64 (0.47–0.87)	.005	0.70 (0.57–0.88)	.002
Age at the first RFA	1.01 (0.99–1.02)	.447	1.01 (1.00–1.02)	.212
Female	1.58 (1.22–2.04)	<.001	1.35 (1.11–1.63)	.003
BMI	1.00 (0.97–1.03)	.969	1.01 (0.99–1.03)	.392
eGFR, mL/min/1.73 m^2^	1.00 (0.99–1.01)	.033	0.99 (0.99–1.00)	.370
Current smoking	1.33 (1.02–1.73)	.033	1.06 (0.87–1.29)	.568
Current drinking	0.79 (0.60–1.05)	.104	0.81 (0.66–1.00)	.048
Persistent AF	1.16 (0.94–1.42)	.174	1.16 (1.00–1.35)	.049
Congestive heart failure	1.12 (0.76–1.65)	.578	0.75 (0.54–1.04)	.081
Hypertension	1.34 (1.07–1.68)	.011	1.14 (0.97–1.35)	.110
Diabetes	0.94 (0.71–1.26)	.694	0.79 (0.63–0.99)	.043
Thromboembolism	1.21 (0.83–1.76)	.317	1.04 (0.78–1.39)	.798
Major bleeding	0.99 (0.57–1.72)	.979	1.00 (0.65–1.52)	.991
Anteroposterior diameter of LA	1.02 (1.00–1.04)	.037	1.02 (1.01–1.04)	.001
Statins	0.86 (0.67–1.11)	.254	0.85 (0.71–1.03)	.094
ACEIs/ARBs	1.15 (0.89–1.49)	.284	1.08 (0.89–1.31)	.448

Abbreviations: ACEI, angiotensin‐converting enzyme inhibitors; AF, atrial fibrillation; ARBs, angiotensin receptor blockers; BMI, body mass index; CI, confidence interval; DAT, diagnosis‐to‐ablation time; eGFR, estimated glomerular filtration rate; HR, hazard ratio; LA, left atrium; RFA, radiofrequency ablation.

^a^ Composite outcomes: cardiovascular death, embolism, major hemorrhages, or cardiac rehospitalization.

Kaplan–Meier estimates of composite outcomes between DAT ≤ 1 year and nonablation are shown in Figure S1. After adjusting potential confounders, DAT ≤ 1 year tended to be, though not statistically significant, associated with a lower composite outcomes risk (DAT ≤ 1 year: HR = 0.78, 95% CI: 0.58–1.05, *p* = .099).

**Figure 2:**
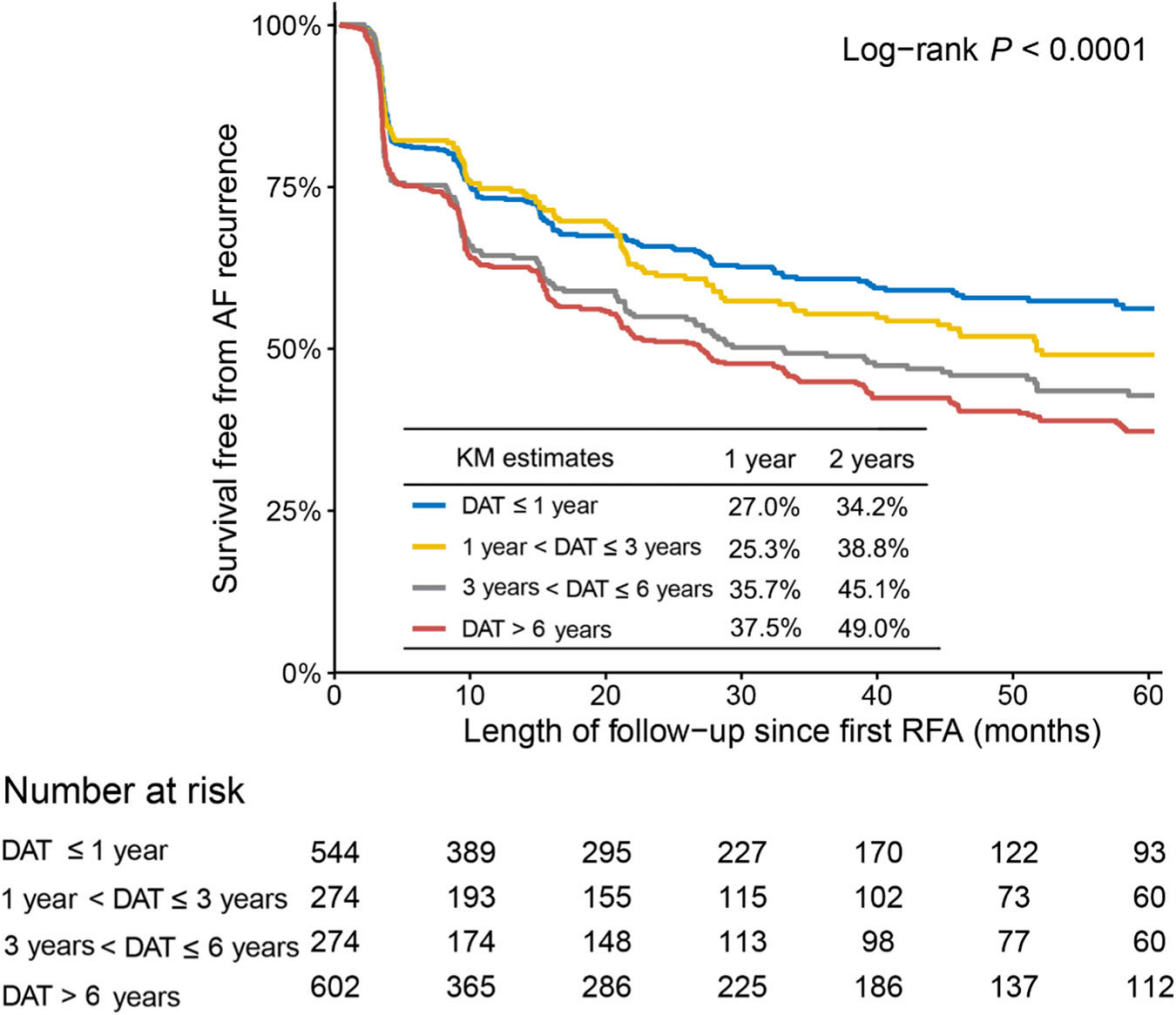
Kaplan–Meier (KM) curves for AF recurrence since the first RFA among patients with early‐onset AF by different DAT intervals. AF, atrial fibrillation; DAT, diagnosis to ablation time; RFA, radiofrequency ablation.

During follow‐ups, if patients experienced any symptom suggesting arrhythmia recurrence would be advised to go to any hospital for examination, and 80.7% of participating patients provided ECG or 24‐h Holter or ILR records (72.3% provided ECG records, and 57.3% provided 24‐h Holter or ILR records), and 815 recurrence cases were identified by cardiologists based on patients' reports and records review. Incidence of AF recurrence since the first RFA increased with the increase of DAT intervals (DAT ≤ 1 year: 16.8 per 100 person‐years, 1 year < DAT ≤ 3 years: 18.4 per 100 person‐years, 3 years < DAT ≤ 6 years: 23.0 per 100 person‐years, DAT > 6 years: 26.8 per 100 person‐years; *p* < .001). Kaplan–Meier estimates of AF recurrence appeared lower in the shorter DAT groups (Figure 2; log‐rank *p* < .001). After adjustment, DAT ≤ 1 year was shown associated with lower AF recurrence risk in comparison with DAT > 6 years group (Table 3; DAT ≤ 1 year: HR = 0.70, 95% CI: 0.57–0.88, *p* = .002; 1 year < DAT ≤ 3 years: HR = 0.80, 95% CI: 0.63–1.01, *p* = .063; 3 years < DAT ≤ 6 years: HR = 0.97, 95% CI: 0.80–1.21, *p* = .816). Compared with nonablation patients, reduced risk of AF recurrence was also shown in the DAT ≤ 1‐year group (Figure S2; log‐rank *p* < .001; DAT ≤ 1year: adjusted HR = 0.46, 95% CI: 0.38–0.55, *p* < .001).

In addition, the proportions of patients receiving antiarrhythmic agents and anticoagulation agents were slightly decreased during the 5‐year follow‐up period among five groups (Figures S3 and S4).

Subgroup analyses were performed across different phenotypes of AF (Tables S1 and S2). In comparison with the DAT > 6 years group, DAT ≤ 1 year was associated with a lower risk of composite outcomes (persistent AF: HR = 0.60, 95% CI: 0.38–0.94, *p* = .027; paroxysmal AF: HR = 0.66, 95% CI: 0.43–1.00, *p* = .051, *p*_interaction_ = .065) and AF recurrence (persistent AF: HR = 0.70, 95% CI: 0.50–0.98, *p* = .039; paroxysmal AF: HR = 0.72, 95% CI: 0.53–0.96, *p* = .025, *p*_interaction_ = .879) among both persistent and paroxysmal AF patients, while 1year < DAT ≤ 3years was only observed related to decreased risk of AF recurrence in persistent AF patients (persistent AF: HR = 0.59, 95% CI: 0.40–0.87, *p* = .008; paroxysmal AF: HR = 0.99, 95% CI: 0.72–1.36, *p* = .960, *p*_interaction_ = .025).

We also conducted sensitivity analysis by stratifying patients in RFA groups according to year of enrollment (2011–2016 and 2017–2020) and CHF or not (Tables S3–S6). Shorter DAT appeared with a lower incidence of composite outcomes and AF recurrence in each stratification, with a maximum follow‐up period of 2 years. Although more patients with persistent AF enrolled in 2017–2020 than in 2011–2016 (44.4% vs. 38.1%), the crude incidence in each group was similar in the two enrolling periods.

There were 16 postoperative complications (0.94%) in all participants, of which 8 were bleeding or hematoma around puncture points (Table S7). The postoperative complication rates were 0.36% (2/544), 0.73% (2/274), 1.46% (4/274), and 1.32% (8/602), respectively. Two patients experienced cardiac tamponade, and one patient experienced acute cerebral infarction in the DAT > 6 years group; however, all these patients survived and recovered well.

## 4 DISCUSSION

In this study, patients with early‐onset AF who underwent RFA within 1 year since the initial diagnosis had lower cardiovascular events (including: cardiovascular death, embolism, major hemorrhages, and cardiac rehospitalizations) and AF recurrence risk after adjustment of common confounders. The associations remained similar after stratified by AF types.

Growing evidence supports that rhythm control could reduce the rate of recurrent AF for patients with symptomatic AF and successful sinus rhythm maintenance could further improve clinical outcomes.^16^ However, since the onset of AF, the irreversible atrial remodeling process gradually worsens the prognosis of AF patients and hampers the effectiveness of therapy. As it is suggested that treating AF early might potentially halt the progression of the self‐perpetuating effect of AF through the structural remodeling of the atria,^17^ the timing of rhythm‐control therapy may also be important. Early rhythm‐control treatment in patients who have experienced recent‐onset AF has shown promising results over the benefits of rhythm control itself.^18^ From observational studies, early rhythm control was reported to reduce the risk of death and rehospitalization rate due to heart failure or other causes.^5^, ^9^, ^10^, ^19^ In accordance with these results, the recent EAST‐AFNET 4 trial also revealed that early rhythm‐control therapy is associated with a lower risk of adverse cardiovascular outcomes.^4^ Among existing rhythm‐control strategies, RFA is considered an effective way that could terminate the electrical disorder and slow down atrial remodeling and has been confirmed to be more effective than AAD therapy in maintaining sinus rhythm.^20^ In our study, patients with DAT ≤ 1year experienced a lower adjusted risk of cardiovascular events and recurrent AF, providing more supportive evidence for early RFA therapy among Chinese patients.

Most importantly, this study focused on a population with early‐onset AF who was diagnosed with AF before 45 years old. Because AF is considered an age‐related disease, which was more prevalent and was more studied among the elderly population,^4^, ^5^, ^6^, ^7^, ^8^, ^9^, ^10^ evidence for treatment decisions was relatively lacking for young patients. Notably, according to the latest national cross‐sectional study,^21^ patients under 50 years old accounted for 36.4% of the total AF incidence in China, implying an unignorable disease burden caused by early‐onset AF. On the other hand, the pathogenesis and underlying mechanisms of early‐onset AF remain incompletely understood. The increasing number of early‐onset AF patients is probably due to worsening lifestyles, earlier development of cardiovascular risk factors, and especially hereditary factors.^3^ There was a hypothesis that early‐onset AF may be an initial manifestation of severe underlying inherited cardiomyopathies or arrhythmia syndromes caused by genetic variants, and several defined loci had already been confirmed to cause AF at a young age.^22^ As a result of the distinct difference with late‐onset AF patients, the cardiologist might hesitate to give interventional therapy, that is, RFA, for young patients at the early stage of the disease. However, it was reported that atrial structural remodeling changes were already significantly present in patients with early‐onset AF and probably had reduced LVEF,^23^ indicating that early‐onset AF might not be as benign as previously thought and timely intervention was required. Although our findings supported that early RFA might benefit this young population in maintaining sinus rhythm and reducing cardiovascular risk along with a low rate of perioperative complications, further confirmatory studies were still needed to provide more solid evidence.

We also explored the influence of AF types on the prognosis of early‐onset AF patients. While previous studies reported that RFA success rate and clinical prognosis were diminished among persistent AF patients compared with paroxysmal AF patients,^24^ we found similar results that a higher risk of recurrence of AF was presented in persistent AF patients than those with paroxysmal AF since the first RFA procedures. This effect might be due to more pronounced electroanatomical changes in persistent AF patients, indicative of a more progressive remodeling process of the atrial substrate.^25^ Statistics showed a progression happens from paroxysmal to persistent or permanent AF with a rate of 8.6%–15% at 1 year and 24.7% at 5 years after the first diagnosis of AF^26^; however, the Atrial Fibrillation Progression Trial (ATTEST) found that early ablation could delay progression from recurrent paroxysmal AF to persistent AF, which was superior to AAD therapy alone,^27^ and from our results, even for patients who had already developed into persistent AF, early RFA might bring more benefits. Furthermore, we found that earlier RFA was associated with a lower risk of composite outcomes and AF recurrence, but this trend was only found at the ablation of 2011–2016. This may be due to the relatively short follow‐up period for patients undergoing surgery in 2017–2020; therefore, only a tiny number of cumulative events for the composite endpoint and AF recurrence, resulted in a less stable estimated event rate and a statistically insignificant difference among the groups.

The reasons why some patients received RFA early, and others delayed performing RFA might be complex. Among RFA participants in our study, the proportion of those with DAT ≤ 1 year increased from 24.4% in the enrollment period of 2011–2016 to 40.6% in 2017–2020, which indicates ablation was not a widely known and widely recommended intervention in earlier years. The number of interventional electrophysiologists across the whole country, who were qualified to perform AF ablation, was also limited during the early years. Recently, more and more evidence has been given to support the effect of RFA in treating AF,^1^ and this intervention becomes more acceptable in both patients and cardiologists.

Besides rhythm‐control therapy, we also observed other factors influencing the prognosis of AF, including female and larger anteroposterior diameters of LA. Although early‐onset AF was more frequently reported in males, females were confronted with a higher risk of cardiovascular events or recurrent AF in this study. And, our also have previously reported female sex might be an independent risk factor of AF recurrence after AF ablation.^28^ Furthermore, Wong et al. found more advanced atrial remodeling in females than males through high‐density electroanatomic mapping, which might partly explain why females were confronted with a higher risk of cardiovascular events or AF recurrence.^29^

## 5 LIMITATIONS

There are several limitations of our study. First, the nature of the observational study design precludes causal inference. Although we adjusted for patient‐level features by multivariate models, there may still be unmeasured confounding and selection bias that cannot be eliminated only using multivariable models. Second, DAT was defined as the time from the first documented AF to the procedural ablation date, but the actual age of onset may precede the first diagnosis. Third, the type of AF was classified according to the time of RFA, and most of the patients enrolled were not newly diagnosed with AF. Our study lacks data to accurately discriminate long‐standing persistent AF and none‐long‐standing persistent AF, mainly because few patients were regularly monitored before enrollment in China‐AF, and it was impossible to specify the exact time of conversion from paroxysmal to persistent AF in this group of patients. Furthermore, although 81.6% of these patients provided ECG or Holter or ILR records during follow‐ups, the document of AF recurrence was mostly by opportunistic screening or was driven by symptoms. Therefore, the AF recurrence may be underestimated.

## 6 CONCLUSION

In summary, although evidence for treatment decisions was relatively lacking for early‐onset AF patients, who were also confronted with disease burden caused by AF, our study complemented previous studies and provided supportive data that early‐onset AF patients might also benefit from early RFA, regardless of AF type.

## CONFLICTS OF INTEREST STATEMENT

Changsheng Ma has received honoraria from Bristol‐Myers Squibb, Pfizer, Johnson & Johnson, Boehringer‐Ingelheim, and Bayer for giving lectures. Jianzeng Dong has received honoraria from Johnson & Johnson for giving lectures. The remaining authors declare no conflict of interest.

## Supporting information

Supporting information.

## Data Availability

The data that support the findings of this study are available on reasonable request from the corresponding author. The data are not publicly available due to privacy or ethical restrictions.
