# Supplementary material for: Impact of diagnosis‐to‐ablation time on clinical outcomes in patients with early‐onset atrial fibrillation
Source: Clin Cardiol. 2023 Dec 6;47(2):e24194. doi: 10.1002/clc.24194 (PMC10823452; doi:10.1002/clc.24194)
Supplement: Supplementary file 1 — Supporting information. [file CLC-47-e24194-s001.docx]

**Supplementary Material**

**Table 1. Adjusted associations of DAT intervals with composite outcomes since the first RFA among patients with early-onset AF, stratified by the phenotype of AF ***

|  | Incidence  (per100 person years) | Adjusted HR  (95% CI) | *P*  value | *P* for  interaction |
| --- | --- | --- | --- | --- |
| Persistent AF |  |  |  |  |
| DAT>6 years | 11.8 | Ref | Ref |  |
| 3 years<DAT≤6 years | 7.99 | 0.70(0.45-1.08) | 0.108 |  |
| 1 year<DAT≤3 years | 8.21 | 0.79(0.48-1.30) | 0.361 |  |
| DAT≤ 1 year | 7.35 | 0.60(0.38-0.94) | 0.027 |  |
| Paroxysmal AF 0.146 | | | | |
| DAT>6 years | 9.45 | Ref | Ref | 0.414 |
| 3 years<DAT≤6 years | 7.20 | 0.81(0.53-1.23) | 0.108 | 0.065 |
| 1 year<DAT≤3 years | 7.69 | 0.92(0.59-1.44) | 0.724 |  |
| DAT≤ 1 year | 5.43 | 0.66(0.43-1.00) | 0.051 |  |

* Composite outcomes: cardiovascular death, embolism, major hemorrhages, or cardiac rehospitalization.

# Adjusted for age at the first RFA, sex, BMI, eGFR, current smoking, current drinking, congestive heart failure, hypertension, diabetes, thromboembolism, major Bleeding, anteroposterior diameter of LA, statins, ACEIs/ARBs.

DAT= diagnosis to ablation time; RFA= radiofrequency ablation; AF= atrial fibrillation; BMI= body mass index; eGFR= estimated glomerular filtration rate; LA= left atrium; ACEI= angiotensin-converting enzyme inhibitors; ARBs= angiotensin receptor blockers.

**Table 2. Adjusted associations of DAT intervals with AF recurrence since the first RFA among patients with early-onset AF, stratified by the phenotype of AF.**

|  | Incidence  (per100 person years) | Adjusted HR  (95% CI) | *P*  value | *P* for  interaction |
| --- | --- | --- | --- | --- |
| Persistent AF |  |  |  |  |
| DAT>6 years | 31.5 | Ref | Ref |  |
| 3 years<DAT≤6 years | 28.5 | 0.97(0.71-1.31) | 0.821 |  |
| 1 year<DAT≤ 3years | 15.4 | 0.59(0.40-0.87) | 0.008 |  |
| DAT≤ 1 year | 19.8 | 0.70(0.50-0.98) | 0.039 |  |
| Paroxysmal AF 0.962 | | | | |
| DAT>6 years | 23.5 | Ref | Ref | 0.025 |
| 3 years<DAT≤6 years | 19.4 | 1.00(0.74-1.35) | 0.988 | 0.879 |
| 1 year<DAT≤3 years | 20.7 | 0.99(0.72-1.36) | 0.960 |  |
| DAT≤ 1 year | 15.4 | 0.72(0.53-0.96) | 0.025 |  |

# Adjusted for age at the first RFA, sex, BMI, eGFR, current smoking, current drinking, congestive heart failure, hypertension, diabetes, thromboembolism, major Bleeding, anteroposterior diameter of LA, statins, ACEIs/ARBs.

DAT= diagnosis to ablation time; RFA= radiofrequency ablation; AF= atrial fibrillation; BMI= body mass index; eGFR= estimated glomerular filtration rate; LA= left atrium; ACEI= angiotensin-converting enzyme inhibitors; ARBs= angiotensin receptor blockers.

**Table 3. Adjusted associations of DAT intervals with composite outcomes since the first RFA among patients with early-onset AF, stratified by year of ablation***

|  | Incidence  (per100 person years) | Adjusted HR  (95% CI) | *P*  value | *P* for  interaction |
| --- | --- | --- | --- | --- |
| 2011-2016 |  |  |  |  |
| DAT>6 years | 12.2 | Ref | Ref |  |
| 3 years<DAT≤6 years | 8.10 | 0.54(0.32-0.89) | 0.016 |  |
| 1 years<DAT≤3 years | 7.31 | 0.53(0.30-0.94) | 0.031 |  |
| DAT≤ 1 year | 7.04 | 0.49(0.29-0.82) | 0.007 |  |
| 2017-2020 0.952 | | | | |
| DAT>6 years | 13.8 | Ref | Ref | 0.633 |
| 3 years<DAT≤6 years | 7.85 | 0.70(0.37-1.34) | 0.287 | 0.952 |
| 1 years<DAT≤3 years | 9.62 | 0.91(0.48-1.74) | 0.785 |  |
| DAT≤ 1 year | 7.78 | 0.71(0.40-1.29) | 0.263 |  |

* Composite outcomes: cardiovascular death, embolism, major hemorrhages, or cardiac rehospitalization.

# Adjusted for age at the first RFA, sex, AF type, BMI, eGFR, current smoking, current drinking, congestive heart failure, hypertension, diabetes, thromboembolism, major Bleeding, anteroposterior diameter of LA, statins, ACEIs/ARBs.

DAT= diagnosis to ablation time; RFA= radiofrequency ablation; AF= atrial fibrillation; BMI= body mass index; eGFR= estimated glomerular filtration rate; LA= left atrium; ACEI= angiotensin-converting enzyme inhibitors; ARBs= angiotensin receptor blockers.

**Table 4. Adjusted associations of DAT intervals with AF recurrence since the first RFA among patients with early-onset AF, stratified by year of ablation.**

|  | Incidence  (per100 person years) | Adjusted HR  (95% CI) | *P*  value | *P* for  interaction |
| --- | --- | --- | --- | --- |
| 2011-2016 |  |  |  |  |
| DAT>6 years | 36.6 | Ref | Ref |  |
| 3 years<DAT≤6 years | 33.4 | 0.91(0.67-1.22) | 0.517 |  |
| 1 years<DAT≤3 years | 23.8 | 0.68(0.48-0.98) | 0.038 |  |
| DAT≤ 1 year | 22.5 | 0.65(0.47-0.90) | 0.010 |  |
| 2017-2020 0.663 | | | | |
| DAT>6 years | 39.1 | Ref | Ref | 0.403 |
| 3 years<DAT≤6 years | 33.6 | 1.16(0.79-1.71) | 0.443 | 0.642 |
| 1 years<DAT≤3 years | 28.5 | 0.98(0.65-1.49) | 0.937 |  |
| DAT≤ 1 year | 24.3 | 0.83(0.57-1.21) | 0.339 |  |

# Adjusted for age at the first RFA, sex, AF type, BMI, eGFR, current smoking, current drinking, congestive heart failure, hypertension, diabetes, thromboembolism, major Bleeding, anteroposterior diameter of LA, statins, ACEIs/ARBs.

DAT= diagnosis to ablation time; RFA= radiofrequency ablation; AF= atrial fibrillation; BMI= body mass index; eGFR= estimated glomerular filtration rate; LA= left atrium; ACEI= angiotensin-converting enzyme inhibitors; ARBs= angiotensin receptor blockers.

**Table 5. Adjusted associations of DAT intervals with composite outcomes since the first RFA among patients with early-onset AF, stratified by CHF or not***

|  | Adjusted HR  (95% CI) | *P*  value |
| --- | --- | --- |
| CHF |  |  |
| DAT>6 years | Ref | Ref |
| 3 years<DAT≤6 years | 0.36(0.09-1.38) | 0.137 |
| 1 years<DAT≤3 years | 0.79(0.25-2.49) | 0.686 |
| DAT≤ 1 year | 0.40(0.13-1.28) | 0.123 |
| No CHF |  |  |
| DAT>6 years | Ref | Ref |
| 3 years<DAT≤6 years | 0.88(0.65-1.19) | 0.403 |
| 1 years<DAT≤3 years | 0.92(0.66-1.29) | 0.647 |
| DAT≤ 1 year | 0.72(0.53-0.99) | 0.041 |

* Composite outcomes: cardiovascular death, embolism, major hemorrhages, or cardiac rehospitalization.

# Adjusted for age at the first RFA, sex, AF type, BMI, eGFR, current smoking, current drinking, congestive heart failure, hypertension, diabetes, thromboembolism, major Bleeding, anteroposterior diameter of LA, statins, ACEIs/ARBs.

DAT= diagnosis to ablation time; CHF= congestive heart failure; RFA= radiofrequency ablation; AF= atrial fibrillation; BMI= body mass index; eGFR= estimated glomerular filtration rate; LA= left atrium; ACEI= angiotensin-converting enzyme inhibitors; ARBs= angiotensin receptor blockers.

**Table 6. Adjusted associations of DAT intervals with AF recurrence since the first RFA among patients with early-onset AF, stratified by CHF or not.**

|  | Adjusted HR  (95% CI) | *P*  value |
| --- | --- | --- |
| CHF |  |  |
| DAT>6 years | Ref | Ref |
| 3 years<DAT≤6 years | 0.65(0.21-1.98) | 0.448 |
| 1 years<DAT≤3 years | 0.62(0.22-1.77) | 0.377 |
| DAT≤ 1 year | 0.42(0.16-1.14) | 0.088 |
| No CHF |  |  |
| DAT>6 years | Ref | Ref |
| 3 years<DAT≤6 years | 1.05(0.85-1.30) | 0.653 |
| 1 years<DAT≤3 years | 0.86(0.67-1.09) | 0.214 |
| DAT≤ 1 year | 0.78(0.63-0.97) | 0.024 |

# Adjusted for age at the first RFA, sex, AF type, BMI, eGFR, current smoking, current drinking, congestive heart failure, hypertension, diabetes, thromboembolism, major Bleeding, anteroposterior diameter of LA, statins, ACEIs/ARBs.

DAT= diagnosis to ablation time; CHF= congestive heart failure; RFA= radiofrequency ablation; AF= atrial fibrillation; BMI= body mass index; eGFR= estimated glomerular filtration rate; LA= left atrium; ACEI= angiotensin-converting enzyme inhibitors; ARBs= angiotensin receptor blockers.

**Table 7. Periprocedural complications of the first RFA among patients with young-onset AF by different intervals of DAT**

|  | Total  (N=1694) | Intervals of DAT | | | |
| --- | --- | --- | --- | --- | --- |
|  |  | DAT<1 year  (N=544) | 1year<DAT≤3 years  (N=274) | 3years<DAT≤6 years  (N=274) | DAT>6 years  (N=602) |
| Total, n (%) | 16 (0.94) | 2 (0.36%) | 2 (0.73%) | 4 (1.46%) | 8 (1.16%) |
| Thromboembolism, n | 3 | 0 | 1 | 0 | 2 |
| Bleeding complications (hematoma, pseudoaneurysm, arteriovenous fistula), n | 8 | 1 | 1 | 3 | 3 |
| Atrioventricular block, n | 0 | 0 | 0 | 0 | 0 |
| Sinus node injury, n | 1 | 0 | 0 | 1 | 0 |
| Cardiac tamponade, n | 2 | 0 | 0 | 0 | 2 |
| Pulmonary vein stenosis, n | 0 | 0 | 0 | 0 | 0 |
| Pneumothorax or Hemopneumothorax, n | 2 | 1 | 0 | 0 | 1 |
| Left atrioesophageal fistula, n | 0 | 0 | 0 | 0 | 0 |

DAT= diagnosis to ablation time; RFA= radiofrequency ablation; AF= atrial fibrillation.

**Figure 1. Kaplan-Meier curves for composite outcomes between DAT≤1year and non-ablation patients***

**
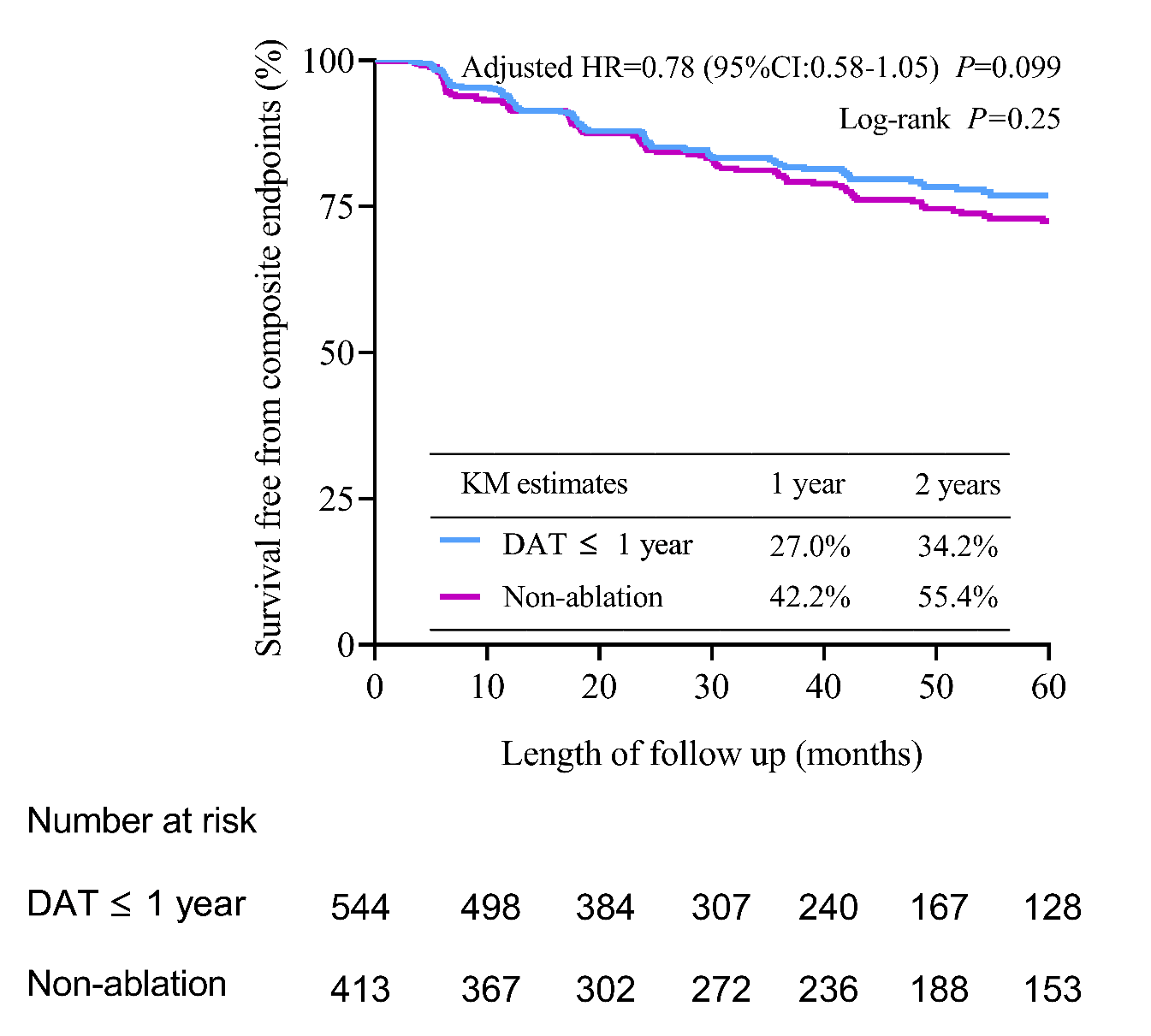
**

# Adjusted for age at the first RFA, sex, AF type, BMI, eGFR, current smoking, current drinking, congestive heart failure, hypertension, diabetes, thromboembolism, major Bleeding, anteroposterior diameter of LA, statins, ACEIs/ARBs.

* Composite outcomes: cardiovascular death, embolism, major hemorrhages, or cardiac rehospitalization.

DAT= diagnosis to ablation time; RFA= radiofrequency ablation; AF= atrial fibrillation.

**Figure 2. Kaplan-Meier curves for AF recurrence between DAT≤ 1 year and non-ablation patients**

**
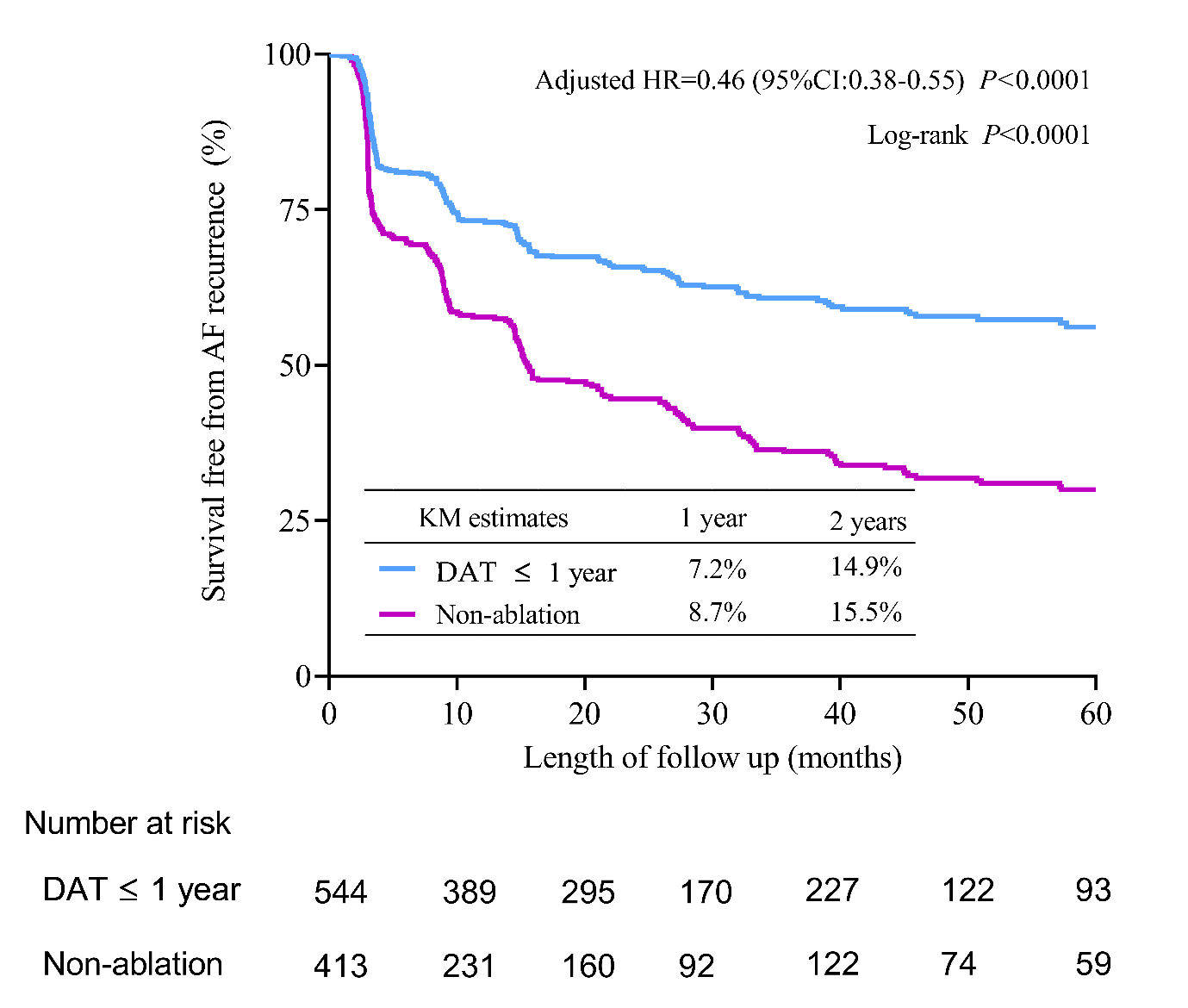
**

# Adjusted for age at the first RFA, sex, AF type, BMI, eGFR, current smoking, current drinking, congestive heart failure, hypertension, diabetes, thromboembolism, major Bleeding, anteroposterior diameter of LA, statins, ACEIs/ARBs.

DAT= diagnosis to ablation time; RFA= radiofrequency ablation; AF= atrial fibrillation.

**Figure 3. The proportion of patients receiving anti-arrhythmic agents during follow-ups**

DAT= diagnosis to ablation time; RFA= radiofrequency ablation.

**Figure 4. The proportion of patients receiving anticoagulant agents during follow-ups**

DAT= diagnosis to ablation time; RFA= radiofrequency ablation.
